# Assessment of Fear of Cancer Recurrence in Patients with Colorectal Cancer and Its Association with Pet Ownership: A Cross-Sectional Study

**DOI:** 10.3390/curroncol32110592

**Published:** 2025-10-23

**Authors:** Enes Erul, Aslı Nur Avcı, Erman Akkus, Ömer Faruk Ayas, Furkan Berk Danısman, Güngör Utkan

**Affiliations:** 1 Department of Medical Oncology, Ankara University Faculty of Medicine, Ankara University, 06590 Ankara, Türkiye; anavci@ankara.edu.tr (A.N.A.); eakkus@ankara.edu.tr (E.A.); utkan@ankara.edu.tr (G.U.); 2 Ankara University Cancer Institute, Ankara University, 06590 Ankara, Türkiye; 3 Department of Internal Medicine, Ankara University Faculty of Medicine, Ankara University, 06590 Ankara, Türkiye; ofayas@ankara.edu.tr; 4 Department of Statistical Sciences, University of Toronto, Toronto, ON M5G 1Z5, Canada; furkan.danisman@mail.utoronto.ca

**Keywords:** colorectal cancer, fear of cancer recurrence, pet ownership, psychosocial oncology, quality of life, survivorship, psychological distress

## Abstract

**Simple Summary:**

Fear that cancer may return is a common and distressing problem for people who have been treated for colorectal cancer. This fear can affect mental health, daily life, and even medical follow-up. While many factors such as sex, education, and mood have been linked to this fear, little is known about the role of pets in supporting patients. In our study of 167 patients, we found that more than half experienced high levels of fear of recurrence. Women and those with anxiety or depression were more vulnerable, while patients with better quality of life reported lower fear. Importantly, owning a pet appeared to protect against this fear, especially in women and patients with smaller families. These findings suggest that pets may offer emotional support and help reduce fear of cancer coming back, opening new possibilities for supportive care and future research.

**Abstract:**

Fear of cancer recurrence (FCR) is a frequent and distressing concern among colorectal cancer (CRC) survivors, often exerting a profound impact on psychological well-being, daily functioning, and treatment adherence. While several clinical and sociodemographic factors have been linked to FCR, the potential role of pet companionship has not been systematically investigated in this population. This cross-sectional study included 167 patients with CRC, assessing FCR with the Fear of Cancer Recurrence Inventory–Short Form (FCRI-SF), psychological distress with the DASS-21, and quality of life with the FACT-G. More than half of the participants (62.3%) met the threshold for high FCR. Multivariable logistic regression revealed that female sex, higher educational attainment, and increased depressive and anxiety symptoms were independently associated with greater odds of high FCR. Conversely, better overall quality of life was linked to lower FCR, with each additional FACT-G point reducing the likelihood of high fear by 5%. Notably, pet ownership emerged as a robust protective factor: pet owners demonstrated approximately one-quarter the odds of high FCR compared with non-owners. Subgroup analyses suggested that this protective effect was particularly evident among women and patients with fewer children, groups potentially more vulnerable to social isolation. These findings highlight pet ownership as a novel factor associated with reduced FCR in CRC patients and suggest potential directions for supportive interventions integrating companion animals into survivorship care.

## 1. Introduction

Colorectal cancer remains one of the most significant global health burdens, with over 1.9 million new diagnoses and approximately 935,000 related deaths reported worldwide in 2020, according to GLOBOCAN data. These figures rank it as the third most commonly diagnosed malignancy and the second leading cause of cancer-related mortality globally [1]. Notably, in recent decades, the incidence of early-onset colorectal cancer (EOCRC)—diagnosed before the age of 50—has shown an alarming increase, the underlying causes of which are still not fully understood. Simultaneously, a shift toward younger age at diagnosis has been observed, with the median age decreasing from 72 to 66 years between 1990 and 2016 [2].

Advancements in cancer care, including more refined surgical techniques, immunotherapies, and molecularly targeted treatments, have contributed to prolonged survival in patients diagnosed with colorectal cancer [3]. As survival rates improve, however, it has become increasingly important to address the long-term physical and emotional challenges patients face. In colorectal cancer survivorship, prominent physical sequelae include bowel dysfunction (e.g., diarrhea/constipation, urgency, fecal incontinence and low anterior resection syndrome), abdominal/pelvic pain and bloating, stoma-related issues, chemotherapy-induced peripheral neuropathy (notably after oxaliplatin), sexual and urinary dysfunction, and fatigue; these frequently co-occur with psychological concerns such as anxiety, depressive symptoms, and fear of cancer recurrence [4,5].

Among these psychosocial concerns, fear of cancer recurrence (FCR) has emerged as one of the most prevalent and distressing experiences reported by cancer survivors. Also referred to in some contexts as fear of progression, FCR refers to the persistent fear, worry, or anticipation that cancer might return or worsen over time. It is now broadly understood as a multifaceted psychological phenomenon, encompassing chronic anxiety, cognitive intrusions related to disease recurrence, hypersensitivity to bodily sensations, and an enduring sense of threat—all of which can substantially interfere with daily functioning [6].

Despite its high prevalence, FCR is not consistently identified or managed in routine oncology care. During outpatient rehabilitation intake visits, roughly one quarter of patients reported not disclosing all of their needs and burdens. Among the concerns left unspoken, fear of cancer recurrence was the most frequently cited (≈45.7%). Patients may not volunteer recurrence-related worries, generic distress screens can miss FCR, and recent work calls for brief FCR-specific screening and clear referral pathways within survivorship services [7,8]. Yet, evidence links high FCR to reduced quality of life, impaired emotional regulation, and poor psychosocial adaptation, it may also manifest in physical symptoms such as fatigue and behavioral consequences such as sleep disturbance, appetite irregularities, and maladaptive coping patterns like hypervigilance and reassurance-seeking [9,10,11]. More severe levels of FCR have even been associated with increased healthcare utilization and system-level costs, as patients may seek repeated medical evaluation or request hospitalization for psychological reassurance [12].

In recent years, interest has grown in complementary approaches aimed at supporting patients’ emotional well-being. Animal-assisted therapies, in particular, have drawn attention for their potential to reduce stress, ease anxiety, promote social connectedness, and foster emotional comfort in chronically ill populations [13]. Beyond formal animal-assisted interventions, everyday pet relationships may plausibly benefit cancer survivors through multiple pathways: affective co-regulation and stress reduction, less loneliness and stronger social connection, restorative routine and light physical activity (particularly among dog owners), and a sustained sense of purpose/being needed despite illness-related changes [14,15]. Qualitative reports also describe non-judgmental companionship, comfort, and a return to normalcy at home. Because FCR closely tracks psychological distress and quality-of-life domains, these mechanisms provide a coherent basis to examine whether pet ownership relates to lower FCR in colorectal cancer [16]. However, to date, the possible relationship between pet ownership and fear of cancer recurrence has not been empirically examined, several gaps remain. Oncology lacks standardized, evidence-based guidance for animal-assisted approaches; existing recommendations for high-risk patients often emphasize pragmatic infection-control and welfare safeguards but rest on limited data [17]. Most studies are small, and concentrated in breast cancer, limiting generalizability across diagnoses, sexes, and cultures [18]. Critically, the relationship between everyday pet ownership and FCR has seldom been evaluated in colorectal cancer. Addressing this gap, we assess FCR across sociodemographic and clinical factors and test whether pet ownership is associated with lower FCR. We hypothesized that pet companionship may buffer recurrence-related anxiety and contribute to reduced FCR severity.

## 2. Materials and Methods

### 2.1. Study Design and Participants

This cross-sectional study was conducted between 6 September 2024, and 3 August 2025, at the Department of Medical Oncology, Ankara University, among participating patients diagnosed with colorectal cancer. Inclusion criteria were as follows: being 18 years of age or older, having a confirmed diagnosis of colorectal cancer, capacity to consent, being fully oriented to person, place, and time, and having the ability to read, write, understand, and communicate in Turkish. Eligibility did not depend on remission status or treatment phase. Participants were enrolled during routine outpatient visits and could be in surveillance/remission, receiving maintenance systemic therapy, or undergoing active treatment at the time of survey completion. No stage I cases were enrolled during the accrual window; thus, the analyzed cohort comprised stages II–IV. Disease stage was abstracted from the medical record, summarized descriptively (Table 1), and included as a covariate in multivariable models (stage III–IV vs. II; Table 4). To address potential confounding, we also recorded time since diagnosis and recurrence status. Patients who did not meet these criteria or had cognitive impairments or communication barriers were excluded from the study.

### 2.2. Data Collection and Measures

Sociodemographic information—including patients’ age, sex, marital status, education level, pet ownership status, and whether they had a caregiver (and the identity of the caregiver, if applicable)—was collected. Additionally, clinical data were recorded, including cancer diagnosis, disease stage, time since diagnosis, and history of recurrence (if any).

#### 2.2.1. Fear of Cancer Recurrence (FCR)

The severity of fear of cancer recurrence was assessed using the Fear of Cancer Recurrence Inventory—Short Form (FCRI-SF) [19]. This self-report instrument consists of 9 items rated on a 5-point Likert scale ranging from 0 (“not at all”) to 4 (“all the time”). The total score is calculated by summing the scores of all items except item 5, as per the original scoring guideline. Possible total scores range from 0 to 36, with higher scores indicating greater severity of FCR. In accordance with prior studies, a cut-off score of ≥13 was used to identify patients with high levels of FCR [20]. The Turkish version of the FCRI-SF has previously been validated, and its reliability and validity have been established in earlier studies [21]. The internal consistency of the FCRI-SF in our sample was excellent, with a Cronbach’s alpha coefficient of 0.896.

#### 2.2.2. Depression Anxiety Stress Scale (DASS-21)

The DASS-21 questionnaire consists of a total of 21 items, divided equally into three subscales: depression, anxiety, and stress, each represented by seven items. All items are scored on a 4-point Likert scale, ranging from 0 (did not apply to me at all) to 3 (applied to me very much or most of the time), reflecting the respondent’s experience over the past week. Scoring is based on the sum of items within each subscale, and previously established cut-off values from the literature were used to interpret symptom severity [22]. The Turkish version of the DASS-21 has previously undergone translation, cultural adaptation, and psychometric validation [23]. The internal consistency of the DASS-21 subscales in this study was excellent, with Cronbach’s alpha coefficients of 0.883 for depression, 0.850 for anxiety, and 0.887 for stress.

#### 2.2.3. Quality of Life (QoL)

Quality of life was assessed using the Functional Assessment of Cancer Therapy—General (FACT-G) scale, which comprises four distinct domains: Physical Well-Being (PWB), Social/Family Well-Being (SWB), Emotional Well-Being (EWB), and Functional Well-Being (FWB). Each item is rated on a 5-point Likert scale ranging from 0 (not at all) to 4 (very much), with negatively worded items reverse-coded during analysis. Domain scores were summed to calculate a total FACT-G score, where higher scores indicate better overall quality of life. The Turkish version of the FACT-G has previously undergone translation, cultural adaptation, and psychometric validation [24]. The FACT-G demonstrated excellent internal consistency in this study, with a Cronbach’s alpha coefficient of 0.901.

#### 2.2.4. Statistical Analysis

Statistical analyses were performed using IBM SPSS Statistics, version 22.0, and multivariate analyses were conducted in RStudio version 2024.12.0 + 467. The reliability of each scale was assessed through Cronbach’s alpha coefficients, as well as by evaluating test–retest stability using Pearson’s correlation analysis and the paired-samples *t*-test. Total and subscale scores of the administered instruments were examined according to sociodemographic and clinical variables. For categorical variables, frequency distributions were reported. To identify the specific groups contributing to observed differences, homogeneity of variance was first evaluated. When this assumption was met, Bonferroni post hoc tests were applied; when violated, Tamhane’s T2 multiple comparison tests were used. Differences between two independent groups were analyzed using the independent-samples *t*-test, whereas comparisons involving more than two independent groups were conducted Via one-way analysis of variance (ANOVA). Associations between two continuous variables were examined with Pearson’s correlation coefficient, while relationships between categorical variables were tested using the chi-square test. Given that the dependent variable was continuous, a linear regression model was constructed using the Enter method to identify independent predictors. For the multivariate analysis, given that the dependent variable was binary, multiple logistic regression models were constructed to identify independent predictors. To further examine the influence of the primary variable of interest, pet ownership, analyses were stratified by relevant subgroups to assess whether its effect varied across different population segments.

A total of 167 patients were included in the final analysis. Post hoc power analysis was conducted using G*Power (v3.1), assuming a medium effect size (Cohen’s d = 0.5), an alpha level of 0.05, and two-tailed testing. The achieved power (1–β) was calculated to be approximately 0.95, indicating that the sample size was sufficient to detect statistically significant differences between groups. Moreover, similar observational studies exploring psychological outcomes in cancer patients have included comparable or smaller sample sizes, further supporting the adequacy of the current cohort size for exploratory and hypothesis-generating purposes [10].

To address potential selection bias, we conducted a sensitivity analysis using stabilized inverse probability of treatment weighting (IPTW). The propensity score was estimated via logistic regression with pet ownership as the dependent variable and the following covariates selected a priori: age, ECOG (0–1 vs. ≥2), Charlson Comorbidity Index (CCI), disease stage (III–IV), time since diagnosis (≤1 year, 1–3 years, >3 years), radiotherapy, surgery, recurrence status, and number of children. Given near-ubiquitous chemotherapy exposure, chemotherapy was excluded from the PS to avoid perfect prediction. Stabilized weights were trimmed at the 1st–99th percentiles. Balance was assessed using standardized mean differences (SMDs), targeting |SMD| < 0.10. The treatment effect on high FCR was then estimated with a weighted binomial model with robust standard errors. Full pre/post-weighting SMDs and weight percentiles are provided in Supplementary Tables S1–S3, and a Love plot is shown in Supplementary Figure S1.

## 3. Results

A total of 167 patients were included in the study. The mean age of participants was 61 years (range: 30–82). Of the total cohort, 17 patients (10.2%) were younger than 50 years, meeting the definition of early-onset colorectal cancer (EOCRC), while 71 patients (42.5%) were aged over 65 years. Of the participants, 61.7% were male, 87.4% were married, and 92.2% had at least one child. An evaluation of patients’ employment status revealed that 58.7% were retired, 12% had never been employed, and 18% had left their jobs due to cancer. Regarding educational attainment, the largest proportion of patients (49.7%) had completed primary or middle school, followed by those with a high school diploma (22.8%) and university graduates (22.2%). The majority of patients (63.5%) reported that their primary caregiver was their spouse, followed by their children in 26.3% of cases. In this cohort, 28 individuals (16.8%) reported pet ownership. Of these, 18 (10.8%) kept cats, 4 (2.3%) kept dogs, 3 (1.8%) kept both a cat and a dog, and 3 (1.8%) owned other types of pets. Exploratorily, among cat-only owners (*n* = 18), 27.8% (5/18) met the high-FCR threshold, compared with 50.0% (2/4) among dog-only owners (Fisher’s exact *p* = 0.56). At diagnosis, 4.2% were stage 2, 52.1% stage 3, and 43.7% stage 4. Recurrence was observed in 15.0% of patients. In 18.0% of the patients, more than one year had passed since their diagnosis, while in 16.8%, the time since diagnosis exceeded three years. Among 167 patients, colon 117 (70.1%) and rectum 50 (29.9%).

The mean FCRI-SF score in the study population was 14.99 (SD = 8.27). Using the cut-off of ≥13 to define high FCR, 62.3% of participants were classified in the high FCR group. In sensitivity analyses using alternative FCRI-SF thresholds, 49.7% met ≥16 and 25.1% met ≥22. Significant differences were observed for sex (*p* = 0.012)—a higher proportion of women had high FCR compared to men. Pet ownership was also significantly associated with lower FCR levels (*p* < 0.001). No statistically significant differences were found for age group, marital status, education, employment status, primary caregiver type, cancer stage, recurrence status, or time since diagnosis. By treatment intent, pet ownership was comparable (curative 16.0% vs. palliative 17.8%; *p* = 0.835). High FCR (≥13) was higher in the palliative group (65.8% vs. 59.6%), but not significant (*p* = 0.426). High FCR (FCRI-SF ≥ 13) occurred in 63.2% of colon vs. 60.0% of rectum cases; the difference was not statistically significant (χ^2^ *p* = 0.692). Including location as a covariate did not materially alter our main findings. More detailed information is provided in Table 1.

**Figure 1:**
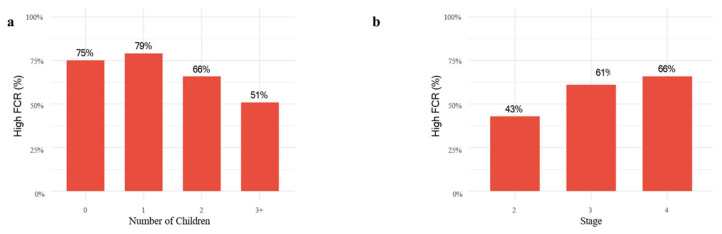
Proportion of patients with high fear of cancer recurrence by (**a**) number of children and (**b**) cancer stage. Bars represent the proportion within each category, with percentages calculated relative to the total group size.

Although children acted as the primary caregivers in only 26.3% of cases, Figure 1a illustrates that the prevalence of high fear of recurrence declined as the number of children increased. In an exploratory analysis including an explicit 0-children group (n = 13), FACT-G Social/Family Well-Being did not differ significantly across 0, 1, 2, and ≥3 children (Kruskal–Wallis *p* ≈ 0.43). In contrast, Figure 1b demonstrates that FCR tended to rise with advancing cancer stage, showing a marked increase from stage II to stage III, but remaining relatively stable between stages III and IV (Figure 1).

Within the DASS-21 subscales, the highest mean score was observed for stress (5.84 ± 4.95), whereas in the FACT-G, social/family well-being (20.01 ± 4.34), emerged as the most preserved domain, followed by functional (16.28 ± 5.41) and emotional well-being (15.16 ± 5.29) (Table 2).

**Figure 2:**
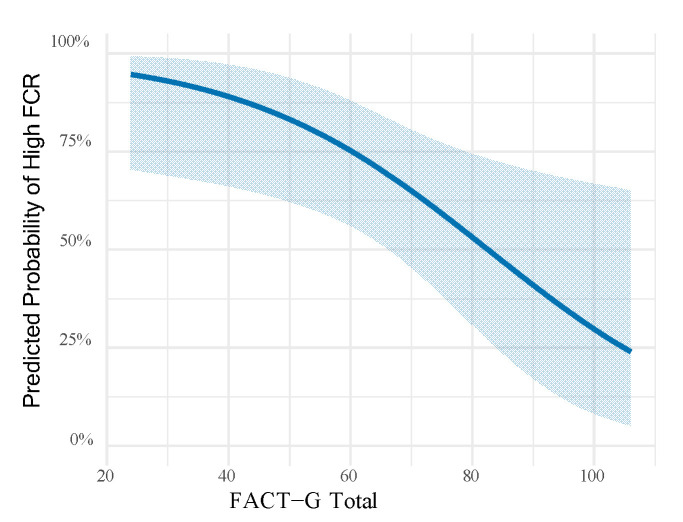
Predicted probability of high fear of cancer recurrence across the range of FACT-G total scores, based on the logistic regression model with all subjects. Shaded areas represent 95% confidence intervals.

FCR showed significant positive correlations with depression (r = 0.547, *p* < 0.001), anxiety (r = 0.569, *p* < 0.001), and stress (r = 0.618, *p* < 0.001) as measured by the DASS-21. Conversely, FCR was negatively correlated with all FACT-G domains, including physical well-being (r = −0.568, *p* < 0.001), emotional well-being (r = −0.589, *p* < 0.001), functional well-being (r = −0.421, *p* < 0.001), and social/family well-being (r = −0.252, *p* = 0.001). Similarly, the total FACT-G score was inversely associated with FCR (r = −0.599, *p* < 0.001) (Table 3). In the multivariable adjusted logistic regression model (Table 4), pet ownership emerged as a strong protective factor against high fear of recurrence, with pet owners showing about one-quarter the odds compared to non-owners (OR = 0.25, 95% CI: 0.08–0.45, *p* = 0.002). Female patients demonstrated significantly greater vulnerability, with nearly four times higher odds of high FCR relative to males (OR = 3.87, 95% CI: 1.80–10.70, *p* = 0.012). Furthermore, each incremental point in the FACT-G total score was associated with a 5% reduction in the likelihood of high fear (OR = 0.95, 95% CI: 0.92–0.98, *p* < 0.001). Notably, higher education levels were also linked to greater odds of experiencing elevated FCR (OR = 1.80, 95% CI: 1.18–2.48, *p* = 0.020) (Table 4). Figure 2 illustrates the influence of life quality in greater detail, showing that higher scores decreases predicted probability of having high fear of recurrence from around 95% to 25%.

**Table 3:** Correlations between FCRI-SF total score and DASS-21/FACT-G scores.

Variable	r	*p*-Value
Depression (DASS-21)	0.547	<0.001
Anxiety (DASS-21)	0.569	<0.001
Stress (DASS-21)	0.618	<0.001
Physical Well-Being (FACT-G)	−0.568	<0.001
Emotional Well-Being (FACT-G)	−0.589	<0.001
Functional Well-Being (FACT-G)	−0.421	<0.001
Social/Family Well-Being (FACT-G)	−0.252	0.001
FACT-G Total	−0.599	<0.001

**Table 4:** Multivariable Adjusted Logistic Regression Analysis of Factors Associated with High Fear of Cancer Recurrence in Patients with Colorectal Cancer.

Variable	OR	95% CI (Lower–Upper)	*p*-Value
Female (vs. Male)	3.87	1.80–10.70	0.012
Depression	1.16	1.04–1.24	<0.001
Anxiety	1.42	1.07–2.05	0.040
Stress	1.01	0.62–1.73	0.273
Education	1.80	1.18–2.48	0.020
FACT-G Total	0.95	0.92–0.98	<0.001
Pet Ownership (Yes)	0.25	0.08–0.45	0.002
Age	0.98	0.95–0.99	0.105
Time since diagnosis	1.01	0.85–1.15	0.626
Stage (III–IV vs. II)	1.42	0.75–2.02	0.170

Notes: OR = Odds Ratio; CI = Confidence Interval.

Patients were divided into two groups: those with up to two children and those with three or more. Logistic regression analyses showed that pet ownership was a significant protective factor only among individuals with fewer children. Within this group, owning a pet reduced the odds of reporting high fear of recurrence by approximately 85% (OR = 0.15). Sex-based analyses further revealed a differential pattern. Among women, pet ownership was strongly associated with lower fear of recurrence (OR = 0.08), corresponding to a reduction of about 92% in odds. No comparable effect was observed among men, indicating that the psychological benefits of pet companionship may be more meaningful for female patients. Finally, subgroup analyses compared predictors between pet owners and non–owners. For patients without pets, factors such as sex, education, and overall quality of life retained their significance. However, in the pet-owner group, these predictors lost their statistical impact, suggesting that pet companionship may attenuate or even override the influence of other established risk factors. (Supplementary Table S5)

For sensivity analyses stabilized weights had a median of 0.969 (IQR 0.895–1.066), with an effective sample size of 159.2. Post-weighting, covariate balance improved substantially (maximum |SMD| decreased from 0.404 pre-weighting to 0.148 post-weighting; most covariates <0.10). The IPTW estimate indicated lower odds of high FCR among pet owners (OR 0.158, 95% CI 0.061–0.408). Balance diagnostics and the distribution of stabilized weights are reported in Supplementary Tables S1–S3 and Figure S1.

## 4. Discussion

Fear of cancer recurrence is a common yet often unmet concern among patients following a diagnosis of colorectal cancer. In our study, we observed that more than half of the participants reported high levels of FCR, underscoring its prevalence as a significant psychological burden in this patient population. However, reports on the prevalence of FCR among colorectal cancer (CRC) survivors have shown considerable variability across studies [25]. These discrepancies are largely attributable to differences in the measurement tools applied and, in the thresholds, used to define “clinically significant” FCR [25]. For example, a meta-analysis in mixed cancer populations estimated that, depending on the FCRI-SF cutoff applied, between 30.0% and 53.9% of patients experienced subclinical or clinical levels of FCR [26]. Specifically, 53.9% were above the ≥ 13 threshold, 43.3% exceeded ≥ 16, and 30.0% met the ≥ 22 criterion [26]. Similarly, a Danish validation study reported that 29% of CRC patients had clinical FCR (defined as FCRI-SF ≥ 16) [27]. In another investigation, 56% of CRC survivors exceeded the widely used cutoff of FCRI-SF > 13, and 18.3% surpassed the stricter threshold of > 22 compatible with our findings [28]. In another study based on clinical psychology assessments, 52% of CRC survivors were reported to experience FCR, with 34% classified as moderate to severe [29]. We also note several case-mix features that could modestly inflate FCR relative to some reports: our sample was drawn from an outpatient medical-oncology setting, included only stage II–IV patients (no stage I accrued during the study window), and had ~65% surveyed ≤1 year from diagnosis—contexts that have been linked to higher distress/FCR in prior work.

Our analysis showed that pet ownership, sex, education, anxiety, depression, and quality of life were significant predictors of fear of recurrence. These findings highlight several key factors influencing fear of cancer recurrence. The sex disparity, with women exhibiting greater fear, is consistent with prior research on psychological distress in oncology and may reflect differences in coping strategies or perceived vulnerability [26,28,30]. The inverse association between quality-of-life scores and fear underscores the importance of overall well-being in mitigating recurrence-related anxiety. In a study with patients with gastric cancer, FCR has been found to be more closely linked to social, psychological, and health-related quality of life factors rather than demographic or socioeconomic variables [31]. Similarly, in another study conducted among patients with colorectal cancer, lower quality of life was associated with higher levels of FCR [25]. Unexpectedly, higher education was associated with increased FRC which could reflect unobserved confounding factors or the possibility that higher education increases patients’ awareness of potential risks, thereby elevating fear levels. In contrast to our results, some studies have indicated that lower educational attainment (such as middle school or below) is linked to an increased risk of FCR [32,33]. By contrast, other research has found no significant association between education level and FCR, underscoring the inconsistencies observed across different populations and methodological approaches [30]. Further research focusing specifically on educational effects could provide deeper insights.

Disease stage and severity have generally been associated with higher levels of FCR in some reports [28] yet other studies in CRC survivorship and mixed-cancer cohorts have found no clear or direct relationship between stage and recurrence-related fear, particularly after accounting for psychological distress and quality-of-life domains [25,34,35,36]. In our data, the proportion of patients with high FCR increased from stage II to stage III but then plateaued between stages III and IV, suggesting a threshold effect whereby crossing a certain clinical severity may heighten perceived threat without further linear escalation thereafter. This pattern aligns with evidence that symptom burden, anxiety/depression, and overall quality of life often explain more variance in FCR than medical variables alone [34,36]. Consistent with that literature, when we adjusted for covariates in multivariable analyses, stage did not remain an independent predictor of high FCR (Table 4), even though patients at advanced stages may understandably report greater fear at a descriptive level due to perceived risks of treatment resistance, recurrence, or progression. Heterogeneity within stage IV (including patients with durable disease control), and the dominant contribution of psychological distress and quality-of-life factors; thus, FCR need not increase linearly with actuarial prognosis at higher disease burden. Collectively, these observations underscore that while disease severity can shape patients’ threat appraisals, psychological and functional well-being are pivotal determinants of FCR and should be proactively addressed in survivorship care [25,34,35,36].

In our cohort, FCR declined as the number of children increased. We view this as evidence of distributed family support rather than reliance on a single caregiver: larger families likely provide more frequent emotional reassurance, companionship, and practical assistance, which can temper threat appraisal and recurrence-related worry. Additional pathways may include greater perceived security, role continuity/feeling needed, and regular social contact, all of which relate to lower distress. This interpretation accords with the inverse association between social/family well-being and FCR observed in our data. We also noted that the association between pet ownership and lower FCR was most apparent among patients with fewer children, a group at higher risk of limited day-to-day support. In this setting, pets may operate as an alternative or supplementary source of emotional support, partially substituting for close-in family resources. Clinically, these findings suggest that patients with smaller family networks may benefit most from pet-inclusive or animal-assisted adjuncts within survivorship care, while recognizing that prospective, adequately powered studies are needed to confirm these effects. Notably, solo living among pet owners in Türkiye is uncommon—approximately 4.1% for dog owners and 7.4% for cat owners—compared with much higher rates in some European samples (e.g., 50.1% and 59.1% in Ireland), consistent with an extended-family model in which pet keeping and caregiving often reflect joint family decisions [37]. The same report indicates that children influenced ~21% of pet-adoption decisions, underscoring the role of family dynamics [37]. This context helps explain why the protective association of pet ownership was most evident in those with fewer children: national data—low solo living among pet owners and a tendency for family-level decision-making—support the view that pets can supplement close-in family support when family networks are smaller.

The relevance of pet ownership for cancer survivors—and its contribution to well-being—derives from combined physical and psychosocial benefits. Companion animals are frequently associated with better quality of life in people living with chronic illness and are increasingly integrated into therapeutic settings. Pets can function as a supportive resource, helping to lower stress, anxiety, and depressive symptoms. Following a cancer diagnosis, this support may attenuate heightened distress. Notably, 80% of breast cancer survivors report that their pets help them feel loved and needed and provide a positive presence at home. Survivors also highlight pets’ non-judgmental response to illness-related changes (e.g., alterations in body image) [18]. Interactions with animals have been linked to favorable neuroendocrine responses—including release of dopamine, oxytocin, prolactin, and endorphins—and survivors commonly describe gains in joy, comfort, and hope [38]. Animal-Assisted Therapy (AAT) has been used to ease distress, normalize hospital environments, and distract from pain, treatment toxicity, or anxiety; oncology patients in one study indicated that pet visits made therapy feel easier and better [39]. In terminal illness, animals’ empathic presence can provide emotional support and soften fear of death; some patients even credit their pets with motivating them to survive during suicidal ideation [40]. Physiologically, contact with animals has been associated with reductions in blood pressure and heart rate [14]. Ownership can also promote greater activity and movement: many survivors report being motivated by their pets to go outdoors and adopt healthy routines such as walking (one breast cancer survivor attributed getting out of bed to her dog’s need to urinate) [40]. Pet ownership has been linked to lower cardiovascular disease risk, and dog ownership has been associated with protection against disability onset in older Japanese adults, with greater benefit when combined with regular exercise [41]. Pets may also aid pain management via distraction or comforting touch, and pet-focused interventions have been proposed to improve treatment adherence by shifting attention away from disease and symptoms [14].

Building on this, survivorship care could incorporate a brief screen-and-support workflow for patients with constrained close-in support. During routine distress assessments, one or two items capturing perceived support/loneliness, together with a brief prompt about current pet relationships or interest in animal-assisted contact, may be included. For individuals who screen positive, low-risk, clinician-mediated adjuncts to standard psycho-oncology—such as certified therapy-animal visits or scheduled, pet-inclusive routines coordinated with a caregiver—may be considered. Safety procedures—including infection-control considerations during immunosuppression, allergy review, and verification of animal welfare/handler certification—should be specified, participation documented in the care plan, and outcomes tracked (e.g., FCRI-SF and mood/quality-of-life measures) to evaluate feasibility and benefit [17]. Such a targeted, protocol-driven approach may enable teams to leverage pet companionship where family networks are limited, without advocating de novo pet acquisition during active treatment.

In this cross-sectional analysis, pet ownership was inversely associated with high FCR after multivariable adjustment and in IPTW sensitivity analyses. These results should be interpreted as associative rather than causal; unmeasured factors (e.g., perceived loneliness, attachment, housing, prior mental health) may influence both the likelihood of keeping a pet and FCR. Prospective designs that measure psychosocial mediators and, where feasible, trials of clinician-mediated animal-assisted contacts are needed to test whether enhancing human–animal connection yields reductions in FCR. To assess generalizability and plausibility, we situate our rates against population-level surveys and clinical cohorts. At the household level in Türkiye, a nationally representative 2024 survey indicates that approximately one-third of households keep any pet, with cat ownership (~13%) exceeding dog ownership (~8%) [42]. Our clinic-based rate (16.8%) is lower—plausibly reflecting older age, comorbidities, treatment-related constraints, and urban housing—yet it aligns with clinical/older-adult cohorts internationally, where current ownership often falls around 14–21% [41,43]. According to the official PETVET registry (January 2025), households with a registered cat or dog constitute approximately 9–10% of all households in Türkiye; because mandatory microchipping/registration began in 2021, this figure likely underestimates true ownership [37]. Taken together, these benchmarks support the contextual consistency of our 16.8% clinic rate and the observed cats > dogs’ pattern, while we acknowledge limited generalizability from a single-center oncology cohort.

Our study has several limitations that should be acknowledged. First, it was conducted in a single center with a relatively limited number of participants, which may restrict the generalizability of the findings. Additionally, selection/non-response bias is possible because non-attendees and non-consenters were not characterized. We mitigated confounding using multivariable adjustment and propensity score weighting, but residual bias cannot be excluded; results should be interpreted as associative and hypothesis-generating. Second, due to its cross-sectional design, causal relationships cannot be established for such a complex phenomenon as fear of cancer recurrence. Another limitation of our study is the inclusion of patients across different disease stages, yielding a heterogeneous cohort. We used number of children as a pragmatic indicator of family network size; however, this is a coarse proxy that does not capture perceived support, loneliness, contact frequency, co-residence, or caregiver intensity. Future studies should incorporate brief, validated psychosocial instruments to delineate these mechanisms more precisely. A further limitation is that we did not systematically classify treatment phase at the time of the survey (surveillance vs. maintenance vs. active therapy), which may contribute to clinical heterogeneity; nevertheless, we adjusted for stage and considered time since diagnosis and recurrence status in our analyses. Finally, one of the major limitations is the limited depth of information regarding the pet ownership variable, which may have reduced the ability to capture nuances such as the type, duration, or quality of pet companionship. Despite these limitations, our study has important strengths. To the best of our knowledge, this is the first investigation to specifically explore the relationship between fear of cancer recurrence and pet ownership in patients with colorectal cancer. In addition, the use of robust statistical analyses and the application of validated and reliable measurement tools strengthen the credibility of our findings and provide a meaningful contribution to the existing literature.

## 5. Conclusions

Our findings demonstrate that fear of recurrence is highly prevalent among patients with colorectal cancer and is influenced by a combination of demographic, psychological, and quality-of-life factors. Importantly, pet ownership emerged as a significant protective element, particularly in subgroups at higher risk of social isolation such as women and individuals with fewer children. While the study is limited by its single-center, cross-sectional design and the relatively simple categorization of pet ownership, it represents the first attempt to explore this relationship in colorectal cancer. The results emphasize the need for broader recognition of FCR in survivorship care and open new avenues for considering companion animals as part of psychosocial support strategies. Future longitudinal and multicenter studies are warranted to clarify causal pathways and to evaluate whether structured interventions involving pets or animal-assisted therapy could meaningfully reduce FCR and enhance quality of life in this vulnerable population.

## Figures and Tables

**Table 1:** Baseline characteristics stratified by pet ownership (owners vs. non-owners).

	Total (n = 167)	Pet Ownership		*p*-Value
		Owners (n = 28)	Non-Owners (n = 139)	
Age (years), median (range)	61 (30–82)	60 (30–78)	63 (33–82)	0.124
Sex				
Male	103 (61.7%)	20 (71.4%)	83 (59.7%)	0.291
Female	64 (38.3%)	8 (28.6%)	56 (40.3%)	
Marital Status				
Married	146 (87.4%)	25 (89.3%)	121 (87.1%)	1.000
Single/Widowed/Divorced	21 (12.6%)	3 (10.7%)	18 (12.9%)	
Education Level				
Primary/Secondary School	83 (49.7%)	12 (42.9%)	71 (51.1%)	0.538
High School	38 (22.8%)	7 (25.0%)	31 (22.3%)	
University and above	37 (22.2%)	6 (21.4%)	31 (22.3%)	
Master’s/Doctorate	9 (5.4%)	3 (10.7%)	6 (4.3%)	
Employment Status				
Currently employed	19 (11.4%)	6 (21.4%)	13 (9.4%)	0.271
Unemployed	20 (12.0%)	2 (7.1%)	18 (12.9%)	
Left job due to cancer	30 (18.0%)	4 (14.3%)	26 (18.7%)	
Retired	98 (58.7%)	16 (57.1%)	82 (59.0%)	
Parental Status				
No children	13 (7.8%)	3 (10.7%)	10 (7.2%)	0.459
Has children	154 (92.2%)	25 (89.3%)	129 (92.8%)	
Primary Caregiver				
Spouse	106 (63.5%)	17 (60.7%)	89 (64.0%)	0.391
Parent(s)	9 (5.4%)	2 (7.1%)	7 (5.0%)	
Child	44 (26.3%)	6 (21.4%)	38 (27.3%)	
Other (Sibling, None, Caregiver)	8 (4.8%)	3 (10.7%)	5 (3.6%)	
Income (per family member)				
≤$1000	106 (63.5%)	15 (53.6%)	91 (65.5%)	0.230
>$1000	61 (36.5%)	13 (46.4%)	48 (34.5%)	
Charlson Comorbidity Index (median, range)	2 (0–5)	2 (0–4)	2 (0–5)	0.052
ECOG Performance status				
0–1	134 (80.2%)	24 (85.7%)	110 (79.1%)	0.591
≥2	33 (19.8%)	4 (14.3%)	29 (20.9%)	
Stage				
Stage 2	7 (4.2%)	0 (0.0%)	7 (5.0%)	0.477
Stage 3	87 (52.1%)	15 (53.6%)	72 (51.8%)	
Stage 4	73 (43.7%)	13 (46.4%)	60 (43.2%)	
Tumour location				
Colon	117 (70.1%)	18 (64.3%)	99 (71.2%)	0.465
Rectum	50 (29.9%)	10 (35.7%)	40 (28.8%)	
Treatment Intent				
Curative	94 (56.3%)	15 (53.6%)	79 (56.8%)	0.835
Palliative	73 (43.7%)	13 (46.4%)	60 (43.2%)	
Treatment type				
Surgery	130 (77.8%)	20 (71.4%)	110 (79.1%)	0.453
Chemotherapy *	160 (95.8%)	28 (100%)	132 (94.9%)	0.603
Radiotherapy ^¶^	59 (35.3%)	11 (39.3%)	48 (34.5%)	0.667
Recurrence status				
No recurrence	142 (85.0%)	24 (85.7%)	118 (84.9%)	1.000
Recurrence	25 (15.0%)	4 (14.3%)	21 (15.1%)	
Time since diagnosis				
Less than 1 year	109 (65.3%)	20 (71.4%)	89 (64.0%)	0.548
1–3 years	30 (18.0%)	3 (10.7%)	27 (19.4%)	
More than 3 years	28 (16.8%)	5 (17.9%)	23 (16.5%)	

* Chemotherapy indicates any receipt during the disease course (adjuvant, neoadjuvant, palliative, or maintenance), not current treatment status. ^¶^ Radiotherapy indicates any RT delivered during the disease course (pre-operative, post-operative/adjuvant, or palliative/metastasis-directed), not current treatment status and not limited to pelvic RT.

**Table 2:** Mean ± SD scores for FCRI-SF, DASS-21, and FACT-G (total and subscales).

Scale/Subscale	Mean ± SD
FCRI-SF Total	14.99 ± 8.27
DASS-21 Depression	5.48 ± 5.13
DASS-21 Anxiety	4.73 ± 4.36
DASS-21 Stress	5.84 ± 4.95
FACT-G Physical Well-Being	18.10 ± 6.22
FACT-G Emotional Well-Being	15.16 ± 5.29
FACT-G Functional Well-Being	16.28 ± 5.41
FACT-G Social/Family Well-Being	20.01 ± 4.34
FACT-G Total	69.53 ± 16.71

## Data Availability

The data presented in this study are available on reasonable request from the corresponding author. The data are not publicly available due to privacy and ethical restrictions.

## Supplementary Materials

The following supporting information can be downloaded at: https://www.mdpi.com/article/10.3390/curroncol32110592/s1, Table S1: Standardized mean differences before and after weighting Table S2: Distribution of stabilized inverse probability weights. Table S3: IPTW-weighted association between pet ownership and high FCR. Table S4: Sociodemographic Characteristics of Patients with Colorectal Cancer Participating in the Study. Table S5: Logistic regression of High FCR: odds ratios (OR) with significance stars. Figure S1: Covariate balance before and after IPTW (Love plot).

## Abbreviations

The following abbreviations are used in this manuscript:

CRC	Colorectal cancer
EOCRC	Early-onset colorectal cancer
FCR	Fear of cancer recurrence
FCRI-SF	Fear of Cancer Recurrence Inventory—Short Form
DASS-21	Depression Anxiety Stress Scale—21
FACT-G	Functional Assessment of Cancer Therapy—General
PWB	Physical Well-Being
SWB	Social/Family Well-Being
EWB	Emotional Well-Being
FWB	Functional Well-Being
IPTW	Inverse Probability of Treatment Weighting
SMD	Standardized Mean Difference
OR	Odds Ratio
CI	Confidence Interval
QoL	Quality of Life
RT	Radiotherapy
PS	Propensity Score
