# Supplementary material for: Assessment of Fear of Cancer Recurrence in Patients with Colorectal Cancer and Its Association with Pet Ownership: A Cross-Sectional Study
Source: Curr Oncol. 2025 Oct 23;32(11):592. doi: 10.3390/curroncol32110592 (PMC12651416; doi:10.3390/curroncol32110592)
Supplement: Supplementary file 1 [file curroncol-32-00592-s001.zip › curroncol-3862734-supplementary.pdf]

Supplementary Figure S1. Covariate balance before and after IPTW (Love plot)

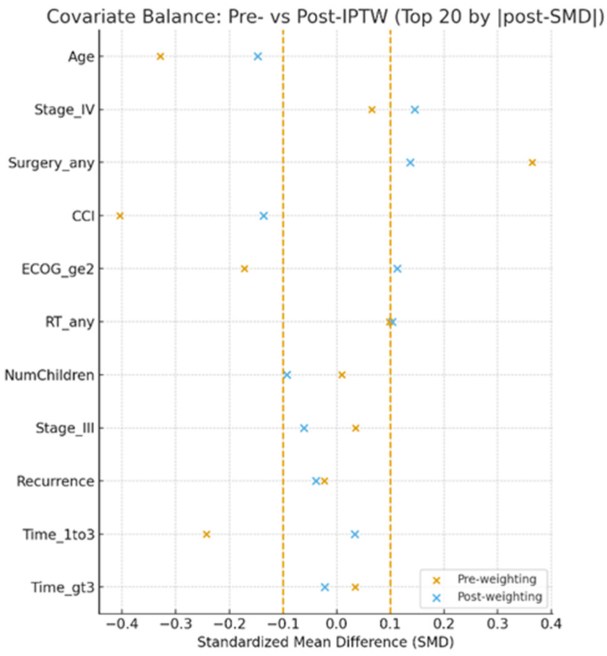

Standardized mean differences (SMDs) for all propensity-score covariates before and after stabilized inverse probability of treatment weighting (IPTW); vertical reference lines at |SMD|=0.10.

Supplementary Table S1. Standardized mean differences before and after weighting

Covariate	SMD_pre	SMD_post
Age	-0.32871	-0.14768
Stage_IV	0.064935	0.145528
Surgery_any	0.363941	0.136413
CCI	-0.40433	-0.13633
ECOG_ge2	-0.17177	0.112912
RT_any	0.09753	0.103473
NumChildren	0.009496	-0.09294
Stage_III	0.035128	-0.06118
Recurrence	-0.02297	-0.03855
Time_1to3	-0.24305	0.033606
Time_gt3	0.034341	-0.02224

Pre- and post-IPTW SMDs for each covariate used in the propensity score.

Supplementary Table S2. Distribution of stabilized inverse probability weights

	stabilized_IPTW
count	167
mean	0.98968378
std	0.220189879
min	0.531936593
1%	0.539909473
5%	0.648052231
25%	0.894871757
50%	0.968739852
75%	1.066298197
95%	1.221852185
99%	2.124634859
max	2.136019975

Percentiles, median, and IQR of stabilized IPTW; includes effective sample size.

Supplementary Table S3. IPTW-weighted association between pet ownership and high FCR

Model	Effect	OR	CI_low	CI_high
IPTW (stabilized)	Pet ownership (vs no pet)	0.158337201	0.061445592	0.408014

Weighted odds ratio (OR) and 95% CI from a binomial model with robust SEs; exposure is pet ownership (yes vs no), outcome is high FCR.

Table S4. Sociodemographic Characteristics of Patients with Colorectal Cancer Participating in the Study

	Total (n= 167)	Fear of cancer recurrence		p-value
		Low (n= 63)	High (n= 104)	
Age (years), median (range)	61 (30-82)	61 (30-78)	61.5 (33-82)	0.916
Sex				
Male	103 (61.7%)	47 (74.6%)	56 (53.8%)	**0.012**
Female	64 (38.3%)	16 (25.4%)	48 (46.2%)	
Marital Status				
Married	146 (87.4%)	54 (85.7%)	92 (88.5%)	0.637
Single/Widowed/Divorced	21 (12.6%)	9 (14.3%)	12 (11.5%)	

Education Level				
Primary/Secondary School	83 (49.7%)	34 (54%)	49 (47.1%)	0.058
High School	38 (22.8%)	12 (19%)	26 (25%)	
University and above	37 (22.2%)	17 (27%)	20 (19.2%)	
Master's / Doctorate	9 (5.4%)	0 (0.0%)	9 (8.7%)	
Employment Status				
Currently employed	19 (11.4%)	7 (11.1%)	12 (11.5%)	0.576
Unemployed	20 (12.0%)	6 (9.5%)	14 (13.5%)	
Left job due to cancer	30 (18.0%)	9 (14.3%)	21 (20.2%)	
Retired	98 (58.7%)	41 (65.1%)	57 (54.8%)	
Parental Status				
No children	13 (7.8%)	4 (6.3%)	9 (8.7%)	0.590
Has children	154 (92.2%)	59 (93.7%)	95 (91.3%)	
Primary Caregiver				
Spouse	106 (63.5%)	39 (61.9%)	67 (64.4%)	0.796
Parent(s)	9 (5.4%)	4 (6.3%)	5 (4.8%)	
Child	44 (26.3%)	18 (28.6%)	26 (25.0%)	
Other (Sibling, None, Caregiver)	8 (4.8%)	2 (3.2%)	6 (5.8%)	
Income (per family member)				
≤ $1000	106 (63.5%)	39 (61.9%)	67 (64.4%)	0.782
> $1000	61 (36.5%)	24 (38.1%)	37 (35.6%)	
Pet Ownership				
No	139 (83.2%)	43 (68.3%)	96 (92.3%)	**<0.001**
Yes	28 (16.8%)	20 (31.7%)	8 (7.7%)	
Charlson Comorbidity Index (median, range)	2 (0–5)	2 (0–4)	2 (0–5)	0.428
ECOG Performance status				
0–1	134 (80.2%)	52 (82.5%)	82 (78.8%)	0.540
≥2	33 (19.8%)	11 (17.5%)	22 (21.2%)	
Stage				
Stage 2	7 (4.2%)	4 (6.3%)	3 (2.9%)	0.457
Stage 3	87 (52.1%)	34 (54.0%)	53 (51.0%)	
Stage 4	73 (43.7%)	25 (39.7%)	48 (46.2%)	
Tumour location				
Colon	117 (70.1%)	43 (68.3%)	74 (71.2%)	0.692
Rectum	50 (29.9%)	20 (31.7%)	30 (28.8%)	
Treatment intent				
Curative	94 (56.3%)	38 (60.3%)	56 (53.8%)	0.426
Palliative	73 (43.7%)	25 (39.7%)	48 (46.2%)	
Treatment type				
Surgery	130 (77.8%)	47 (74.6%)	83 (79.8%)	0.732
Chemotherapy	160 (95.8%)	60 (95.2%)	100 (96.2%)	0.904
Radiotherapy	59 (35.3%)	22 (34.9%)	37 (35.6%)	0.512
Recurrence status				
No recurrence	142 (85.0%)	57 (90.5%)	85 (81.7%)	0.125
Recurrence	25 (15.0%)	6 (9.5%)	19 (18.3%)	
Time since diagnosis				
Less than 1 year	109 (65.3%)	41 (65.1%)	68 (65.4%)	0.978
1–3 years	30 (18.0%)	11 (17.5%)	19 (18.3%)	
More than 3 years	28 (16.8%)	11 (17.5%)	17 (16.3%)	

Table S5: Logistic regression of High FOR: odds ratios (OR) with significance stars

	All	Child = 0,1,2	Child = 3+	Male	Female	Doesn't own pet	Owns pet
Intercept	15.20	97.10	5.79	5.07	1946.00	83.90*	0.00175
Age	0.98	0.95	0.99	0.99	0.97	0.99	0.95
Female	**3.87*** **	1.80	**10.70** **	–	–	**3.52** **	0.34
Depression	1.16	1.04	1.24	0.90	0.51	1.20	0.33
Education	**1.80** **	**1.87***	2.48	**1.88** **	1.43	**1.88** **	2.74
Anxiety	1.42	**2.05***	1.07	**2.02***	0.69	1.24	2.69
Stress	1.01	0.62	1.73	0.77	10.50*	0.96	7.11*
FACT-G total	**0.95** **	0.95	0.95	**0.95***	**0.92** **	**0.93*** **	1.06
Pet ownership	**0.25** **	**0.15** **	0.24	0.45	**0.08***	–	–
Child count = 1	1.19	–	–	–	–	–	–
Child count = 2	0.82	–	–	–	–	–	–
Child count = 3+	0.74	–	–	–	–	–	–
Time since diagnosis	1.01	1.08	0.85	1.15	0.75	–	–
Stage	1.42	2.02	1.43	1.31	1.44	–	–

Notes: Entries are odds ratios. Significance: *** $p < 0.01$, ** $p < 0.05$, * $p < 0.10$.
